# Mild phenotype of *CHAT*-associated congenital myasthenic syndrome: case series

**DOI:** 10.3389/fped.2024.1280394

**Published:** 2024-01-18

**Authors:** Aysylu Murtazina, Artem Borovikov, Andrey Marakhonov, Artem Sharkov, Inna Sharkova, Alena Mirzoyan, Sviatlana Kulikova, Ralina Ganieva, Viktoriia Zabnenkova, Oksana Ryzhkova, Sergey Nikitin, Elena Dadali, Sergey Kutsev

**Affiliations:** ^1^ Research Centre for Medical Genetics, Moscow, Russia; ^2^ Veltischev Research and Clinical Institute for Pediatrics of the Pirogov Russian National Research Medical University, Moscow, Russia; ^3^ Genomed Ltd., Moscow, Russia; ^4^ Republican Research and Clinical Center of Neurology and Neurosurgery, Minsk, Belarus

**Keywords:** *CHAT*, congenital myasthenic syndrome, CMS, mild phenotype, case report

## Abstract

Congenital myasthenic syndrome with episodic apnea is associated with pathogenic variants in the *CHAT* gene. While respiratory disorders and oculomotor findings are commonly reported in affected individuals, a subset of patients only present with muscle weakness and/or ptosis but not apneic crises. In this case series, we describe five individuals with exercise intolerance caused by single nucleotide variants in the *CHAT* gene. The age of onset ranged from 1 to 2.5 years, and all patients exhibited a fluctuating course of congenital myasthenic syndrome without disease progression over several years. Notably, these patients maintained a normal neurological status, except for the presence of abnormal fatigability in their leg muscles following prolonged physical activity. We conducted a modified protocol of repetitive nerve stimulation on the peroneal nerve, revealing an increased decrement in amplitude and area of compound muscle action potentials of the tibialis anterior muscle after 15–20 min of exercise. Treatment with 3,4-diaminopyridine showed clear improvement in two children, while one patient experienced severe adverse effects and is currently receiving a combination of Salbutamol Syrup and pyridostigmine with slight positive effects. Based on our findings and previous cases of early childhood onset with muscle fatigability as the sole manifestation, we propose the existence of a mild phenotype characterized by the absence of apneic episodes.

## 1 Introduction

Congenital myasthenic syndromes (CMSs) comprise a diverse group of hereditary neuromuscular junction disorders characterized by exercise intolerance and muscle weakness that worsens with fatigue. Some CMSs manifest with ocular and bulbar symptoms, such as ptosis, ophthalmoplegia, and respiratory disturbances. Currently, 35 genes have been associated with CMS (1). Congenital myasthenic syndromes (CMSs) can be classified based on various features, encompassing the type of inheritance (autosomal dominant, autosomal recessive), the location of the mutant protein (presynaptic, synaptic, postsynaptic, abnormal glycosylation of acetylcholine receptor subunits), the primary clinical presentation (limb-girdle, ocular), the response to acetylcholine inhibitors, and the long-term course (progressive, fluctuating, regressive) (2, 3).

Pathogenic variants in the *CHAT* gene are associated with CMS with episodic apnea (CMS-EA), also known as CMS type 6. The first case of CMS-EA was published 20 years ago (4), and since then, approximately 50 patients with CMS-EA have been reported (3, 5). The *CHAT* gene encodes choline acetyltransferase, an enzyme involved in the resynthesis of acetylcholine in the nerve terminal (6, 7). CMS-EA is an autosomal-recessive presynaptic disorder, with most known variants in the *CHAT* gene being missense variants (8).

The majority of reported patients with CMS-EA exhibit respiratory disorders and/or oculomotor findings (9). Typically, CMS-EA presents in infancy, characterized by apnea or respiratory insufficiency. However, there have been only a few reported cases without apneic crises, and only one case without both respiratory and oculomotor disturbances (10–14).

In this study, we present five patients from four families with compound heterozygous variants in the *CHAT* gene. Remarkably, all patients exhibit a mild phenotype of CMS without respiratory signs or oculomotor weakness.

## 2 Subjects and methods

Between 2019 and 2022, genetic testing confirmed a diagnosis of *CHAT*-associated CMS in five children (four boys and one girl) from four families. All parents provided informed consent for their children's participation in the study.

The patients were diagnosed at the age of 7–15 years, while the manifestation of the disease occurred at 1–2.5 years of age. Two siblings reside in Belarus, while the remaining three unrelated patients are Russian, residing in different regions of Russia.

Neurological examinations and electrophysiological studies were conducted on all patients (Dantec Keypoint G2, Denmark and Neuro-MEP-micro, Neurosoft, Ivanovo, Russia). The electrophysiological studies included nerve conduction studies and repetitive nerve stimulation (RNS). Standard electrodiagnostic techniques were employed, involving single or repetitive supramaximal nerve stimulation. RNS was performed using a 3 Hz stimulation of the facial nerve (with registration from the orbicularis oculi muscle) and axillary nerve (deltoid muscle) with a 20 s muscle activation period. In addition, a modified 3 Hz RNS technique was applied to the peroneal nerve (tibialis anterior muscle). This procedure was performed immediately following a 15 min session of voluntary physical exercises, with a subsequent RNS applied at the 2–4 min of the rest period. The physical exercises consisted of activities such as walking, running, and stair climbing.

All patients underwent next-generation sequencing (NGS), including either whole-exome sequencing (WES) or whole-genome sequencing (WGS). The WES analysis was carried out using paired-end sequencing (2 × 150 bp) on an IlluminaNextSeq 500 sequencer (Illumina, San Diego, California, U.S.). The library was constructed using the KAPA Hyper Prep Kit (F. Hoffmann-La Roche Ltd, Switzerland). Target enrichment was performed with an IDT xGen® Exome Research Panel v.1 solution capture array (IDT inc., USA), including the coding regions of 19,396 known genes. The detected variants were annotated according to the HGVS (Human Genome Variation Society) nomenclature: https://varnomen.hgvs.org/recommendations/DNA/ (version 20.05) (HGVS, RRID:SCR_012989). The sequencing data were analyzed using the NGS-data-Genome program developed at the Department of Bioinformatics of FSBSI RCMG (registration number 2021662113). Mean coverage was ×76.1, with 0.68% of fragments with less than ×10 coverage. The following predictive algorithms to analyze the pathogenicity of the variants were used: SIFT (Sort Intolerant from Tolerant Human Protein) (SIFT, RRID:SCR_012813) (15), DEOGEN2 (16), MVP (17), and MutationAssesor (18). The detected variants were classified according to the ACMG/AMP guidelines (19). Whole exome sequencing was performed using the equipment of the Genome Shared Use Center (FSBSI RCMG, Moscow, Russia).

WGS was performed using a DNBSEQ-G400 instrument in a pair-ended mode (2 × 150 b.p.) with an average on-target coverage of 30× with MGIEasy FS PCR-Free DNA Library Prep Set (BGI, Beijing, China) for library preparation (Genomed Ltd., Moscow, Russia). Bioinformatic analysis was performed using an in-house software pipeline as described earlier (20) with modifications. In brief, it included quality control of raw reads (FastQC tool v. 0.11.5) followed by read mapping to the hg19 human genome assembly (minimap2 v.2.24-r1122), sorting of the alignments, and marking duplicates (Picard Toolkit v. 2.18.14). Base recalibration and variant calling were performed with GATK3.8. Variant annotation was done using Annovar tool (v.2018Apr16). CNV and SV analysis was performed using Manta tool (v. 1.6.0). Further filtering was performed by functional consequences and population frequencies according to the ACMG recommendations as well as clinical relevance determined by Human Phenotype Ontology database (21).

Segregation analysis and validation of all detected variants were performed by Sanger sequencing carried out using ABIPrism 3,500 Genetic Analyzer (Applied Biosystems, Foster City, CA, USA) according to the manufacturer’s protocol. Primer sequences were designed and variants were named according to the NM_020549.5 transcript variant.

## 3 Case descriptions

Five patients from four unrelated families were diagnosed with *CHAT*-associated myasthenic syndrome. A total of six different single nucleotide variants (SNVs) were identified through NGS studies, with two of them being previously reported. Among these variants, one is a nonsense variant, while the others are missense variants. Three SNVs are classified as pathogenic or likely pathogenic, while the remaining three SNVs are categorized as variants of uncertain significance (Supplementary Table S1).

The first case is a 7-year-old boy (1.1) who presented with episodic weakness in his leg muscles. The proband is the first child of healthy, non-consanguineous Russian parents. The pregnancy and delivery proceeded without complications, and early motor development was unremarkable. The initial episode of severe muscle weakness occurred at the age of 2.5 years while the patient was swimming in a lake with cool water and was unable to exit independently due to muscle weakness. Subsequently, episodes of moderate to severe weakness in the leg muscles would typically occur after walking a distance of around 500 meters. In some instances, the weakness would worsen up to an ambulatory loss. However, these episodes lasted no longer than a few minutes, and muscle strength fully recovered after resting. No other complaints or symptoms were reported. Neurological examination revealed no signs of muscle weakness or fatigability after 2–3 min of exercise or after 10–15 squats. No other neurological abnormalities were detected. Initially, the patient was referred for gene panel sequencing associated with periodic paralysis, but no SNVs were identified. Subsequently, whole-genome sequencing (WGS) was conducted, which revealed two SNVs in the *CHAT* gene (NM_020549.5). One of the SNVs was a previously reported nonsense variant c.451C>T, p.(Arg151Ter) (22, 23), and the other was a novel missense variant c.404C>G, p.(Pro135Arg). Validation and segregation analysis confirmed the compound heterozygous state of the detected SNVs. The treatment with pyridostigmine did not yield a positive response. Further outcome information is unavailable due to loss of communication with proband's parents.

The second patient (proband 2.1) diagnosed with *CHAT*-associated CMS is an 8-year-old boy who experiences similar complaints of severe leg muscle fatigability after walking for 15 min. The proband was born at 39 weeks of gestation from a normal first pregnancy, with a normal height and weight. The parents first noticed symptoms when the boy was 2 years old, as he frequently complained of fatigue while walking. The disease has not progressed, and for several years, it has manifested only with this one symptom. A routine clinical examination revealed a normal neurological status, with no signs of facial or limb muscle weakness during rest or after 5 min of exercises. WGS identified two heterozygous SNVs in the *CHAT* gene: c.404C>G, p.(Pro135Arg) and c.1156T>C, p.(Cys386Arg). Both variants are novel. Segregation analysis confirmed that each parent is an asymptomatic carrier of one of these variants. Notably, a different substitution at the amino acid position c.403C>T, p.(Pro135Ser) has previously been submitted in ClinVar database as a variant of uncertain significance in patient with gait disturbances and muscle weakness. The patient was given 3,4-diaminopyridine at a daily dose of 15 mg, resulting in severe adverse effects, including blurred vision and generalized tonic-clonic seizures. AChE inhibitor therapy (pyridostigmine 180 mg/day) demonstrated insufficient effectiveness. Consequently, Salbutamol Syrup (2 mg/day) was added to the treatment, exhibiting a slight positive effect by increasing the patient's walking distance.

Two male siblings (3.1 and 3.2) exhibited different disease onset and clinical presentations. The older brother experienced a waddling gait and frequent falls starting from the age of 1 year when he began walking independently. These symptoms persisted until around 3–4 years of age. Subsequently, the gait disturbance occurred episodically following physical activity, and the disease stabilized for several years. At the age of 12 years, the patient noticed improvement due to adapting to physical limitations and adopting an appropriate daily activity regimen. Currently, the patient experiences milder symptoms, primarily in the form of transient fatigue. The most recent neurological examination was conducted at the age of 15 years, revealing pathological muscle fatigue only after 35 squats, resulting in a decrease in proximal leg muscle strength to 4/5 (MRC scale). The second boy (3.2) demonstrated increased fatigability during normal physical activities compared to children of the same age, starting at 2 years old. The parents also observed changes in gait following prolonged walking. Both siblings experienced worsening of symptoms during the cold winter season. The younger boy underwent examination at the age of 10 years. After performing repetitive leg lifts and head lifts in the prone position (20 times and 25 times, respectively), a decrease in leg and neck muscle strength to 3/5 was observed. WES was conducted for the younger brother, revealing two SNVs in the *CHAT* gene: one previously reported variant c.1061C>T, p.(Thr354Met) (24–26) and one novel variant c.1841C>G, p.(Ala614Gly). Both SNVs were also identified in the older brother. Each parent is an asymptomatic carrier of one of these variants. AChE inhibitor therapy showed no positive response in both children. Subsequently, they received a dosage of 40 mg/day of 3,4-diaminopyridine and demonstrated clear improvement, as evidenced by an increase in the distance walked without experiencing fatigue.

The fifth proband is an 11-year-old girl who experiences episodic weakness in her leg muscles associated with physical exercises or walking. Fatigability typically occurs after walking approximately 1 km, and muscle strength is restored within several minutes of rest. The disease initially manifested at the age of 1.5 years and is characterized by a gait change while walking. Since then, no new symptoms have occurred, and the disease has shown no signs of progression. Neurological examination revealed decreased strength in the thigh and lower leg muscles only after 15 min of walking and climbing stairs. Muscle weakness following exercise resulted in a waddling gait and Gowers sign when standing up from the floor. The upper limb muscles remain unaffected, and no other neurological abnormalities were detected. Through WES, two single nucleotide variants (SNVs) in a heterozygous state were identified in the *CHAT* gene: c.1061C>T, p.(Thr354Met), and c.971T>G, p.(Leu324Arg). Sanger sequencing was performed for the proband and her parents and confirmed the compound heterozygous state of these SNVs. Pyridostigmine treatment was initiated, but no apparent positive effects were observed within the first 2 weeks.

**Figure 1:**
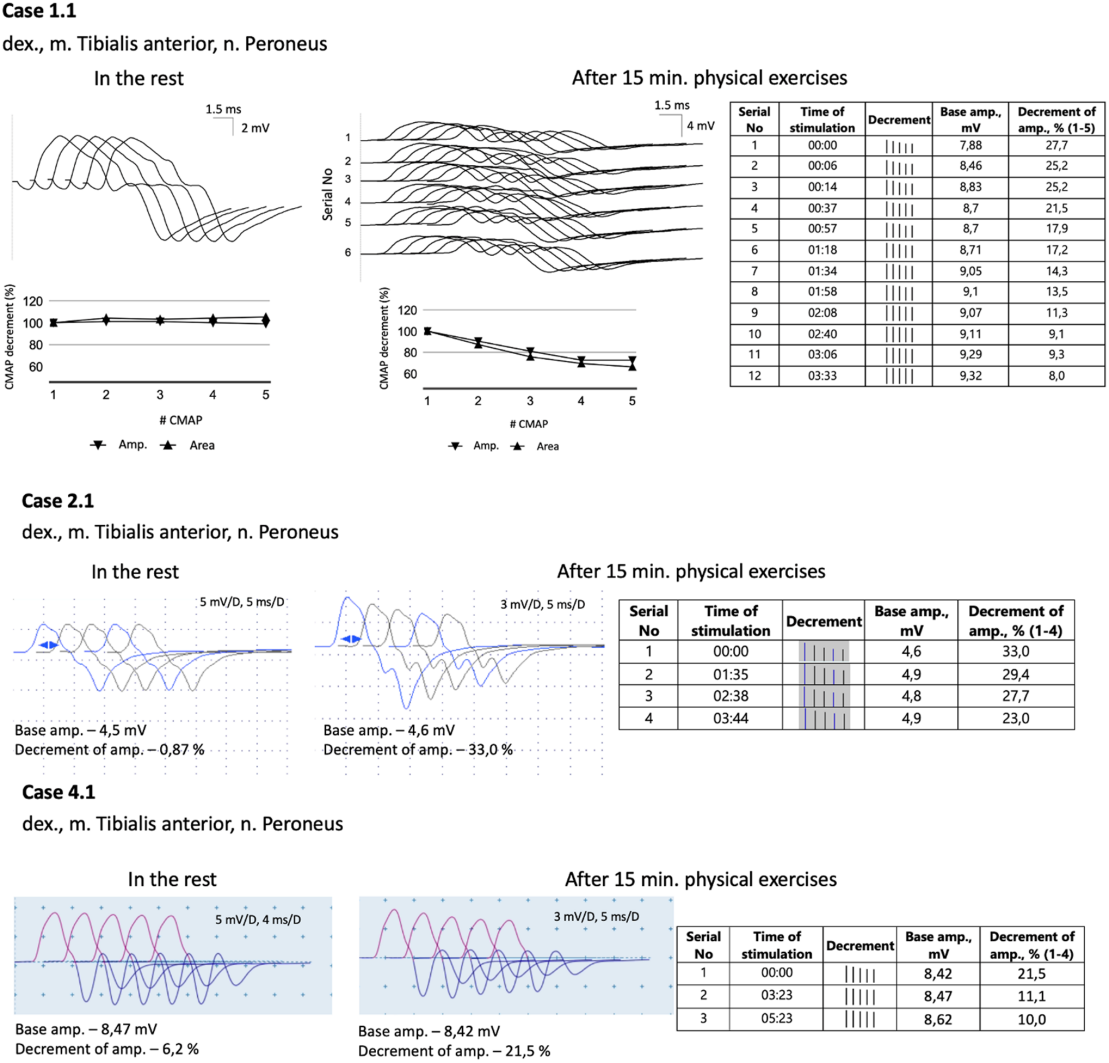
A modified 3 Hz RNS technique applied to the peroneal nerve with CMAPs registration from tibialis anterior muscle. This procedure was carried out after 15 min of physical exercises, which included walking, running, and stair climbing. Subsequently, a 3 Hz RNS was performed after a rest period of 2–5 min.

All five probands underwent nerve conduction studies and RNS of the facial, ulnar, and peroneal nerves. The compound muscle action potentials (CMAP) in all patients exhibited normal amplitude and unchanged form. Standard 3 Hz RNS with 20 s of muscle exercise revealed no abnormalities in all patients. However, considering the development of weakness in the legs after 15–20 min of walking, running, or climbing stairs, the RNS protocol was modified. The RNS study was performed on the peroneal nerve with CMAP registration from the tibialis anterior muscle after 15 min of voluntary muscle activation (Figure 1). In all patients, an increased decrement in amplitude and area of negative CMAP, ranging from 22% to 37%, was detected. This decrement normalized after a rest period of 3–5 min.

## 4 Discussion

We present a case series of five individuals with *CHAT*-associated CMS who exhibited solely exercise intolerance in their leg muscles. Our analysis included a comprehensive review of the literature, identifying a total of 42 cases with clinical descriptions (Table 1). Among these cases, one patient presented with isolated muscle fatigability (10), while an additional five patients demonstrated muscle weakness and ptosis but did not experience apneic crises (11–14). Notably, previously published cases have reported a more severe disease course, characterized by episodic or permanent respiratory disturbances (4, 9–13, 24, 25–35). In nine patients, tracheostomy was necessary to provide respiratory support. The majority of the reported patients (90%, *n* = 38) had disease onset in infancy, with the latest reported onset occurring at the age of four years in a single case report (10). The initial symptoms displayed considerable variation, with four patients presenting isolated exercise intolerance, while most cases exhibited a combination of apnea, hypotonia, and general weakness.

**Table 1:** The representation of clinical signs in previously published and current cases.

Clinical data	Previously published cases	Current cases
Age at examination, years (range, median)	0.2–26 (5)	7–15 (10)
Age of onset, years (range, median)	0–4 (0)	1–2.5 (2)
Initial symptoms		
Exercise intolerance	24% (10/42)	5/5
Ptosis	38% (16/42)	0/5
Facial weakness	14% (6/42)	0/5
Motor development delay	5% (2/42)	0/5
Hypotonia	28% (12/42)	0/5
Respiratory insufficiency	48% (20/42)	0/5
Apnea	28% (12/42)	0/5
Bulbar weakness	28% (12/42)	0/5
Proximal weakness	2% (1/42)	0/5
General weakness	21% (9/42)	0/5
Apneic crises	86% (36/42)	0/5
Ventilation	63% (26/41)	0/5
Tracheostomy	25% (9/36)	0/5
Ptosis	88% (36/41)	0/5
Strabismus or ophtalmoparesis	23% (9/39)	0/5
General muscle weakness	45% (17/38)	0/5
Proximal muscle weakness	67% (26/39)	0/5
Wheelchair dependency	25% (7/28)	0/5
Fatigable leg muscle weakness	96% (27/28)	5/5
Psychomotor delay	57% (21/37)	0/5
Repetitive nerve stimulation		
↑decrement at 3 Hz (20 s ex.)	44% (14/32)	0/5
↑decrement at 10 Hz for 5'	90% (9/10)	n/d
↑decrement at 3 Hz (15 min. ex.)	n/d	5/5
Treatment		
Response to AChE-inhibitor	73% (27/37)	1/5^a^
Response to 3,4-DAP	71% (5/7)	2/3

N/D, no data; ↑, increased; HZ, hertz; SEC., seconds; MIN., minutes; EX., exercises; AChE, acetylcholinesterase; 3,4-DAP, 3,4-diaminopyridine.

^a^ In combination with Salbutamol.

In our cohort of five patients, exercise intolerance was the initial and sole symptom observed (Table 1). The age of onset ranged between 1 and 2.5 years. Importantly, all patients consistently demonstrated a normal neurological status, with the sole exception being the presence of abnormal muscle fatigability subsequent to prolonged physical activity. None of our patients presented with ptosis or ophthalmoparesis, which is in contrast to earlier reported cases where 88% of patients demonstrated ocular symptoms (Table 1).

All five of our patients exhibited a fluctuating course of CMS without progression over the course of several years. Long-term follow-up studies focusing on patients with *CHAT*-associated CMS have been conducted (11, 27). Schara et al. proposed the existence of two distinct phenotypes based on age of onset and clinical progression: neonatal onset and manifestation in early infancy (11). The first group of patients experiences an extremely severe clinical course, with some individuals presenting seizures and intellectual disability. Fatal outcomes during infancy due to *CHAT*-associated CMS have also been documented (11, 28). However, patients with early infantile manifestation often show symptom improvement and a reduction in the number of respiratory crises (11, 27). Considering our patients and additional cases previously described with onset in early childhood and solely presenting muscle fatigability as the single sign of the disease, we propose the existence of a third mild phenotype characterized by the absence of apneic episodes.

Three out of the five patients in our cohort experienced symptom worsening in exposure to cold. This phenomenon has been previously described in five patients with *CHAT*-associated CMS (12, 13, 28). The presence of this symptom is unique to *CHAT*-associated CMS when compared to other forms of CMS and can serve as a valuable clinical clue for diagnosis (28). It is possible that symptom worsening in the cold is more common in *CHAT*-associated CMS, but patients or their parents may not pay adequate attention to it, or it may be rarely detected due to the onset of the disease in infancy in the majority of patients. Studies in Drosophila have demonstrated a significant reduction in ChAT activity and impairment of synaptic transmission, which could explain the exacerbation of symptoms in the cold in patients with *CHAT*-associated CMS (36).

Diverse results of RNS studies in *CHAT*-associated CMS have been reported in the literature. Only a small percentage of patients show a decrement of more than 10% during standard 3 Hz nerve stimulation (11, 12, 27–29). As a result, alternative RNS methods have been proposed and utilized for patients with CMS. One commonly employed method is prolonged subtetanic repetitive nerve stimulation at 10 Hz for 5 min, which has yielded positive results in 9 out of 10 patients (Table 1). However, this method and single-fiber electromyography can be rather painful, especially for children. In light of these considerations, we decided to modify the 3 Hz RNS protocol by conducting it after 15 min of physical exercises. Since all the children in our study exhibited weakness in their leg muscles, we chose to register CMAP with tibialis anterior muscle in response to peroneal nerve stimulation. Immediately following the exercises, we observed an increased decrement of up to 22%–37%, which improved significantly within 3–4 min of rest. Thus, we found this modification of RNS to be informative and applicable even for young children.

The management of *CHAT*-associated CMS presents challenges with regard to treatment. Acetylcholinesterase inhibitors (AChE inhibitors) are typically the first-line therapy, resulting in positive outcomes for approximately 73% of patients (Table 1). However, in cases where there is a limited or absent response, the combination of 3,4-diaminopyridine with AChE inhibitors is considered (11). Within our cohort, only one patient exhibited a mild positive response to treatment with AChE inhibitors in combination with Salbutamol Syrup. Other our patients demonstrated no apparent positive effects from AChE inhibitors therapy, potentially attributed to the delayed initiation of treatment in all cases (ranging from 5 to 14 years from age of onset). Two patients demonstrated significant symptom improvement when treated with 3,4-diaminopyridine. All our patients exhibited a mild disease course since childhood without obvious signs of pronounced disease progression. This leads us to cautiously predict a favorable prognosis for patients. However, the scarcity of cases depicting a mild phenotype makes it challenging to definitively ascertain the long-term prognosis for such patients.

Four novel missense SNVs were identified in our study. Three of these SNVs are currently classified as variants of unknown significance, while one SNV [c.404C>G, p.(Pro135Arg)] is considered likely pathogenic due to a different missense change detected at the same position. In two of our families, a previously reported missense variant [c.1061C>T, p.(Thr354Met)] was found which has been associated with a severe course of CMS characterized by onset in infancy and episodic apneic crises in several patients (24–26). Interestingly, we also observed a nonsense variant that has not been previously reported in patients with CMS but has been documented in individuals with conotruncal defect (transposition of the great arteries) in a heterozygous state with *de novo* origin (22, 23). No second variant in the *CHAT* gene was identified in the two patients with the cardiac phenotype. However, no cardiac defects were observed in our patient with this variant or in their relatives.

**Figure 2:**
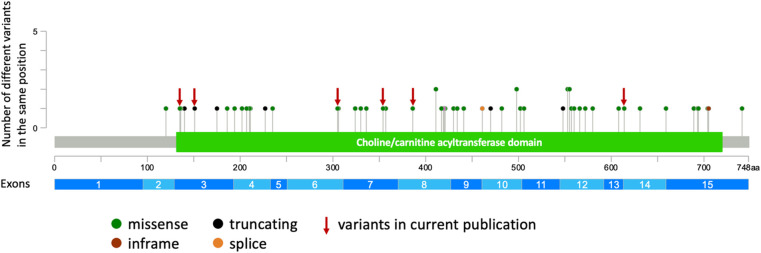
The representation of all described and novel variants in the *CHAT* gene (generated using cBioPortal https://www.cbioportal.org/mutation_mapper).

Establishing precise phenotype-genotype correlations in autosomal recessive diseases presents challenges due to the genotypic heterogeneity within patient cohorts. Numerous cohort studies have been conducted to explore genotype-phenotype correlations in *CHAT*-associated CMS (11, 12, 30). CHAT is an enzyme composed of binding and catalytic domains, with an interfacial active-site tunnel (37, 38). Previous studies have attempted to establish a correlation between the severity of clinical manifestations and the proximity of the variant to the substrate binding and catalytic sites, revealing phenotype-genotype correlations for specific residues (12, 30). Notably, variants in our cohort are uniformly distributed across the entire gene (Figure 2). Contrary to expectations, our analysis revealed that patients sharing the same genotype (e.g., homozygous for I336T) exhibit a broad spectrum of clinical severity (Supplementary Table S2). Furthermore, our observations suggest that loss-of-function variants in the *CHAT* gene may not be exclusively associated with a severe disease course. This inference is supported by both our cohort and previously published cases, where individuals with these variants manifest both mild and severe phenotypes of the disease. To gain a more comprehensive understanding, further functional analysis of novel variants in the *CHAT* gene is imperative, particularly focusing on cases exhibiting a milder disease course that has been identified.

In conclusion, our findings add to the existing literature, which includes six previously published cases without respiratory disorders, and highlight the existence of a milder variant of *CHAT*-associated CMS. This subgroup of patients predominantly presents with abnormal muscle fatigue as the sole or predominant sign, distinguishing them from the more severe presentations reported earlier. Additionally, we introduce a modified RNS protocol that is specifically tailored for pediatric patients with CMS. This modified protocol offers an informative and reproducible approach for assessing neuromuscular function in this patient population.

## Data Availability

The raw data supporting the conclusions of this article will be made available by the authors, without undue reservation.
