# Supplementary material for: Mild phenotype of CHAT-associated congenital myasthenic syndrome: case series
Source: Front Pediatr. 2024 Jan 18;12:1280394. doi: 10.3389/fped.2024.1280394 (PMC10830679; doi:10.3389/fped.2024.1280394)
Supplement: Supplementary file 2 [file Table2.docx]

**SUPPLEMENTAL TABLE 2.** Clinical features of current cases and earlier reported patients.

*Among the previously reported cases, more severe cases are found in the lower rows of the table. Orange cells indicate the presence of positive clinical signs, green cells represent current cases, and gray cells encompass data from previously reported patients without apneic crises. Nonsense or frameshift variants are highlighted in red. СС, current case; F, female; M, male; Y, year; MO, month; INF, infancy; EX INT, exercise intolerance; HP, hypotonia; FW, facial weakness; BW, bulbar weakness; GW, general muscle weakness; PW, proximal muscle weakness; RESP INS, respiratory insufficiency; N/D, no data.*

| **N** | **References** | **Genotype** | **Gender** | **Age at examination** | **Age of onset** | **Initial symptoms** | **Apneic crises** | **Ventilation** | **Tracheotomy** | **Ptosis** | **Strabismus or ophthalmoparesis** | **General muscle weakness** | **Proximal muscle weakness** | **Wheelchair dependency** | **Fatigable leg muscle weakness** | **Psychomotor delay** |
| --- | --- | --- | --- | --- | --- | --- | --- | --- | --- | --- | --- | --- | --- | --- | --- | --- |
| **Total (%)** | | | | | | | **76** | **56** | **22** | **80** | **20** | **39** | **59** | **21** | **97** | **50** |
| 1 | СС 1.1 | P135R; R151X | m | 7 y | 2,5 y | ex int | - | - | - | - | - | - | - | - | + | - |
| 2 | СС 2.1 | P135R; C386R | m | 8 y | 2 y | ex int | - | - | - | - | - | - | - | - | + | - |
| 3 | СС 3.1 | T354M; A614G | m | 15 y | 1 y | ex int | - | - | - | - | - | - | - | - | + | - |
| 4 | СС 3.2 | T354M; A614G | m | 10 y | 2 y | ex int | - | - | - | - | - | - | - | - | + | - |
| 5 | СС 4.1 | L324R; T354M | f | 11 y | 1,5 y | ex int | - | - | - | - | - | - | - | - | + | - |
| **Previously reported cases (%)** | | | | | | | **86** | **63** | **25** | **88** | **23** | **45** | **67** | **25** | **96** | **57** |
| 6 | [10] | G411A; G434S | f | 26 y | 4 y | ex int | - | - | - | - | - | - | - | - | + | - |
| 7 | [12] | R186W; R566C | f | 18 y | 1,5 y | ex int | - | - | - | + | + | - | + | - | + | + |
| 8 | [11] | I336T; I336T | f | 5 y | 1 y | ptosis, ex int, fw | - | - | - | + | + | - | - | - | + | - |
| 9 | [11] | L210P; S498L | f | 12 y | 2 y | ptosis, ex int, gw | - | - | - | + | - | + | + | - | + | - |
| 10 | [13] | V306L; S704del | m | 4 y | inf | motor development delay | - | - | - | + | - | - | - | - | + | + |
| 11 | [14] | V136M; I336T | f | 3 y | inf | ptosis, gw | - | - | - | + | + | + | + | - | + | - |
| 12 | [29] | I336T; I336T | f | 11 y | 1 y | crises with resp ins | + | - | - | - | - | - | - | - | + | - |
| 13 | [28] | L210P;P211L | f | 16 y | n/d | ex int | + | - | - | - | - | - | - | - | + | - |
| 14 | [28] | L210P;P211L | m | 14 y | inf | crises with resp ins | + | - | - | - | - | - | - | - | + | + |
| 15 | [28] | V194L; V506L | f | 8 y | 1,5 y | ex int | + | - | - | + | + | - | + | - | + | + |
| 16 | [11] | V506L; T553dup | f | 1,6 y | inf | ptosis, fw, ex int, crises with resp ins | + | - | - | + | - | - | - | n/d | + | - |
| 17 | [13] | V306L; S704del | m | 9 y | inf | motor development delay | + | - | - | + | - | - | - | - | + | + |
| 18 | [4] | L210P; S498L | f | 6 y | inf | resp ins | + | - | - | + | - | - | - | - | + | - |
| 19 | [28] | S694C; R548X | f | 3 y | inf | crises with resp ins | + | - | - | + | - | - | + | n/d | + | - |
| 20 | [9] | I336T; I336T | f | 1 y | inf | apnea | + | - | - | + | - | + | + | - | + | - |
| 21 | [11] | I336T; I336T | f | 7 y | 1 y | ptosis, pw | + | + | - | + | - | - | + | + | n/d | - |
| 22 | [11] | I336T; I336T | m | 14 y | 1,7 y | ptosis, apnea, hp, fw, ex int, crises with resp ins | + | + | - | + | - | - | + | - | + | + |
| 23 | [11] | I336T; I336T | f | 12 y | 2 y | ptosis, apnea, hp, gw, crises with resp ins | + | + | - | + | - | + | + | + | n/d | + |
| 24 | [29] | I336T; I336T | f | 6 y | 2 y | ptosis, ex int | + | + | - | + | - | - | - | - | + | - |
| 25 | [30] | V136M; S572T | m | 13 y | inf | apnea | + | + | n/d | - | - | n/d | n/d | n/d | n/d | + |
| 26 | [27] | A557T; R548X | n/d | 12 y | inf | hp, ptosis, bw | + | n/d | n/d | + | + | n/d | + | n/d | n/d | + |
| 27 | [11] | I336T; I336T | m | 5 y | inf | ptosis, hp, fw | + | + | - | + | - | - | + | - | n/d | + |
| 28 | [10] | G417A; L502P | m | 7 mo | inf | resp ins, bw | + | + | n/d | + | - | - | - | n/d | n/d | - |
| 29 | [11] | G417R; G417R | m | 5 y | inf | ptosis, apnea, hp, bw | + | + | + | + | - | - | + | + | n/d | + |
| 30 | [11] | I693S; G227Mfs*4 | m | 2 y | inf | ptosis, apnea, hp, bw, crises with resp ins | + | + | + | + | - | - | + | - | n/d | + |
| 31 | [11] | R482G; R482G | m | 5 y | inf | ptosis, ex int, gw, crises with resp ins | + | + | - | + | - | + | + | - | + | + |
| 32 | [11] | I336T; I336T | m | 3 y | inf | ptosis, apnea, hp, resp ins | + | + | + | + | - | - | - | - | - | + |
| 33 | [12] | R207H; R207H | m | 4 y | inf | hp, gw, bw, resp ins | + | + | + | + | + | + | + | + | n/d | + |
| 34 | [12] | R207H; R207H | f | 5 y | inf | gw, fw, bw, resp ins | + | + | + | + | + | + | + | + | n/d | + |
| 35 | [12] | V136M; V136M | f | 8 y | inf | gw, bw, resp ins | + | + | + | + | + | + | + | + | n/d | n/d |
| 36 | [31] | I305T; del ex 4-6 | m | 10 mo | inf | fw, bw, crises with resp ins | + | + | - | + | - | + | + | n/d | + | n/d |
| 37 | [24] | T354M; S694C | m | 1,6 y | inf | hp, resp ins | + | + | - | + | - | + | + | n/d | + | + |
| 38 | [24] | T354M; S694C | f | 2 y | inf | hp, resp ins | + | + | - | + | - | + | + | n/d | + | - |
| 39 | [32] | I336T; I336T | m | 7 y | inf | crises with resp ins | + | + | - | + | - | + | + | - | + | + |
| 40 | [29] | I336T; I336T | m | 13 y | inf | bw | + | + | - | + | - | + | + | + | + | - |
| 41 | [33] | T553N; S704P | f | 3 y | inf | ptosis, hp, bw, resp ins, apnea | + | + | + | + | - | + | + | n/d | n/d | n/d |
| 42 | [26] | T354M; A557T | f | 3 y | inf | apnea | + | + | - | + | - | - | - | - | + | - |
| 43 | [30] | V136M; M202A | f | 2 mo | inf | apnea | + | + | n/d | n/d | n/d | n/d | n/d | n/d | n/d | n/d |
| 44 | [30] | T553N; S704P | f | 3 y | inf | apnea | + | + | n/d | + | n/d | n/d | n/d | n/d | + | + |
| 45 | [34] | I336T; I336T | f | 5 mo | inf | apnea, gw | + | + | n/d | + | - | + | + | n/d | + | + |
| 46 | [35] | F580C; R470X | n/d | 8 y | inf | hp, ptosis, bw | + | + | + | + | + | + | + | n/d | + | + |
| 47 | [36] | R207G; T354M | f | 1 y | inf | resp ins, bw, gw | + | + | + | + | n/d | + | + | n/d | n/d | n/d |
